# The impact of aging on CD4^+^ T cell responses to influenza infection

**DOI:** 10.1007/s10522-018-9754-8

**Published:** 2018-04-03

**Authors:** Erica C. Lorenzo, Jenna M. Bartley, Laura Haynes

**Affiliations:** UConn Center on Aging and Department of Immunology, University of Connecticut School of Medicine, Farmington, CT 06030 USA

**Keywords:** CD4 T cells, Subset differentiation, Influenza, Aging

## Abstract

CD4^+^ T cells are important for generating high quality and robust immune responses to influenza infection. Immunosenescence that occurs with aging, however, compromises the ability of CD4^+^ T cells to differentiate into functional subsets resulting in a multitude of dysregulated responses namely, delayed viral clearance and prolonged inflammation leading to increased pathology. Current research employing animal models and human subjects has provided new insights into the description and mechanisms of age-related CD4^+^ T cell changes. In this review, we will discuss the consequences of aging on CD4^+^ T cell differentiation and function and how this influences the initial CD4^+^ T cell effector responses to influenza infection. Understanding these age-related alterations will aid in the pharmacological development of therapeutic treatments and improved vaccination strategies for the vulnerable elderly population.

## Introduction

The intricacies of aging impact many components of the immune system rendering individuals more susceptible to bacterial and viral pathogens. Not surprisingly, morbidity and mortality from infectious diseases is greatly increased with age; influenza (flu) and pneumonia being among the top killers of people over 65 years of age in the U.S. (Heron 2013). Flu is very problematic for the elderly with over 90% of flu-related deaths occurring in this population (Thompson et al. 2003). While flu-related deaths can be caused by many factors, age-related declines in immune function contribute significantly and lead to multiple age-related manifestations such as delayed and reduced signaling and cytokine secretion by CD4^+^ T cells in response to flu antigens (Garcia and Miller 2001). This, in turn, results in diminished cellular and humoral immunity including reduced B cell responses and defective germinal center formation, as well as dysregulated orchestration and recruitment of inflammatory cells that aid in viral clearance and recovery (Ginaldi et al. 1999; Lefebvre et al. 2016a). CD4^+^ T cells are also essential for robust CD8^+^ T cells responses, crucial for flu viral clearance. It has been demonstrated using transgenic mouse models that depletion of CD4^+^ T cells results in a reduction in the recruitment of CD8^+^ T cells and even further, delayed viral clearance as demonstrated in flu studies (Beuneu et al. 2006; Castellino et al. 2006; Riberdy et al. 2000; Allan et al. 1990).

CD4^+^ and CD8^+^ T cells are appreciated for their diverse T cell receptor repertoire that allows for recognition of epitopes on any given pathogen. Experimental studies, however, have shown this repertoire diversity significantly deceases with age (Johnson et al. 2012; Naylor et al. 2005). Moreover, the frequency of naïve CD4^+^ T cells decreases as the frequency of memory CD4^+^ T cells increases in the periphery, further reducing the ability of aged CD4^+^ T cells in mice and humans to respond to new pathogens (Moro-Garcia et al. 2013). Thymic involution plays a role in the population dynamics of CD4^+^ T cells, however, there are differences in the maintenance of peripheral CD4^+^ T cells between mice and humans. The peripheral diversity of CD4^+^ T cells in mice is more dependent on thymic output, whereas human CD4^+^ T cell diversity is instead heavily dependent on peripheral maintenance (den Braber et al. 2012). In addition, mouse studies have shown the prolonged life-span of naïve CD4^+^ T cells itself could cause intrinsic defects. A reduction in the levels of Bim, a pro-apoptotic protein in the Bcl family, correspond with apoptotic resistance and mediate their longer lifespan in the periphery (Tsukamoto et al. 2010). Although this contributes to overall CD4^+^ T cell homeostasis, longer lived naïve CD4^+^ T cells demonstrate functional defects including reduced proliferation and IL-2 production (Tsukamoto et al. 2009). These age-related decrements result in poor CD4^+^ cell responses to flu infection.

Since CD4^+^ T cells are necessary for robust humoral and cell-mediated flu responses, research investigating the rate and magnitude of these age-related changes throughout the course of infection is of great significance. In order to properly study the magnitude of the response to flu, the primary organs involved must be studied including the lymphoid organs and lungs. As a result, human research examining CD4^+^ T cell deficits during flu infection has some important caveats. First, it is often difficult to obtain samples other than peripheral blood from live young and aged individuals, which limits the ability to examine differences that occur within the lymphoid organs and lungs. Second, it is difficult to determine the initial time and peak of infection and corresponding CD4^+^ T cell responses when only peripheral blood is available. Third, although we can obtain much information from peripheral blood CD4^+^ T cells, and in fact, most studies used to determine clinical outcomes and response to vaccination have been performed using peripheral blood CD4^+^ T cells, it is not always clear if peripheral responses are directly related to the CD4^+^ T cell response within the tissues.

Only recently have researchers begun to examine these early time points and the full kinetics of flu infection in young adults who volunteer to be infected with the flu (Park et al. 2018). This research model, however, is certainly unethical to utilize in the study of the vulnerable aged population who may not be able to survive the course of infection. Thus, the clinical importance of these human studies may not be completely applicable to the elderly population due to the multitude of changes that occur to the immune system with aging. This leaves the majority of human research regarding CD4^+^ T cell changes during flu infection focusing on peripheral blood CD4^+^ T cells during flu recovery or using vaccination responses as predictive markers for infection responses. Although mouse and human CD4^+^ T cell responses exhibit some inherent differences, mouse models have been employed to gain a more in depth understanding of age-related changes within the CD4^+^ T cell compartment. Studies have revealed both cell intrinsic and extrinsic factors that contribute to age-related decrements in CD4^+^ T cell function within lymphoid organs and infected lung tissue (Maue et al. 2009). Here, we describe the significance of the different CD4^+^ T cell subsets during flu infection and how they are altered with aging. Understanding the impact of age-related changes in CD4^+^ T cells and other components of the adaptive immune system will aid in developing better pharmacological and medical interventions and treatment of flu infection in older populations.

## Magnitude of CD4^+^ T cell responses after influenza infection

### Differentiation and Function of CD4^+^ T cell subsets following influenza infection

CD4^+^ T cell differentiation and functional quality is an important component for efficient clearance of flu virus from the lungs. Early mouse studies demonstrated the complexity of this response using CD4^+^ T cell depleting antibodies as well as MHC Class II knockout mice (Topham et al. 1996; Allan et al. 1990). In these models, there is a delay in viral clearance in the absence of CD4^+^ T cells. Others have also shown a reduction in necessary inflammatory mediators and in the numbers of cytotoxic CD8^+^ T cells present at the infection site in the absence of CD4^+^ T cells (Mozdzanowska et al. 1997; Mozdzanowska et al. 2000; Wells et al. 1981; Bourgeois et al. 2002). Taken together, this suggests that CD4^+^ T cells in the lung during flu infection contribute to the cytokine milieu necessary to promote and enhance cellular immunity and efficient clearance of the virus. Furthermore, it has been shown more recently, that without CD4^+^ T cells there is a reduction in germinal center formation (Brown et al. 2004) as well as antibody production from B cells (Mozdzanowska et al. 2005). Indeed, CD4^+^ T cells have functions that promote both cellular as well as humoral immunity (Luckheeram et al. 2012).

While there are numerous subsets of CD4^+^ T cells whose functions are key in various types of immunity, there are several effector types that have been especially implicated in the initial effector response to influenza and viral clearance, as shown in Table 1. Initially, the CD4^+^ T cell compartment is comprised predominantly of a naïve population that in young mice rapidly proliferates and differentiates into appropriate antigen-specific effector subsets (Lanzer et al. 2014). This is largely supported by thymic output of positively selected self-tolerant CD4^+^ T cells. In young mice, the peak of CD4^+^ T cell numbers in the lung occurs just before flu viral clearance and results from effective priming by antigen presenting cells (APCs) leading to expansion of virus-specific effectors (Lefebvre et al. 2012). The flu-specific population consists of CD4^+^ T cells from all of the subsets described in Table 1 throughout the course of infection, highlighting the fact that these uniquely specialized subsets work collectively to carefully regulate the response to flu (Lefebvre et al. 2016b).

**Table 1:** Important subsets of CD4^+^ T cells that mediate regulation and suppression of influenza responses in the lung and draining lymph node

CD4^+^ T cell subset	Role in influenza immunity	
	Function	Cytokine/molecule secretion
Naïve, T_0_	Differentiates into various subsets dependent on cytokine milieu and environmental responses following recognition of viral peptide antigens presented on MHCII molecules by APCs	–
Cytotoxic, T_H_CTL	Responds directly to virally infected cells via MHCII dependent mechanism involving Fas/Fas ligand mediated apoptosis and cytotoxic granule exocytosis	Granzyme B and Perforin
Type 1 helper, T_H_1	Promotes activation of CD8^+^ T cells, macrophages, non-hematopoietic lung epithelial cells	IFN-γ, IL-2, and TNF-α
T follicular helper, T_FH_	Promotes germinal center formation in lymph nodes, B cell differentiation, and high-affinity antibody generation	IL-4 and IL-21
Regulatory, T_reg_	Maintains homeostasis of lung mucosal environment and dampens inflammatory response, necessary for resolution and healing after viral clearance	IL-10 and TGF-β
Memory, T_mem_	Responds more rapidly than naïve CD4^+^ T cells to secondary challenge or to infection following vaccination and differentiates into various subsets dependent on cytokine milieu and environmental responses	–

*APC* antigen presenting cell, *MHCII* major histocompatibility complex class II

Differentiation of naïve CD4^+^ T cells residing in the draining lymph node into the various antigen-specific effector subsets is dependent on presentation of viral antigens via MHC Class II on APCs bearing cognate antigen, cytokines, and environmental and cellular cues. T follicular helper cells (T_FH_) CD4^+^ T cells downregulate the chemokine receptor CCR7 and begin to express markers such as programmed cell death-1 (PD-1), chemokine receptor CXCR5, inducible co-stimulator (ICOS), and the transcription factor B-cell lymphoma 6 protein (BCL6) (Choi et al. 2011; Crotty et al. 2010; Eto et al. 2011; Johnston et al. 2009). This allows for entry into the B cell follicles in order to ultimately promote the generation of high affinity antibodies (Haynes 2008; Hardtke et al. 2005).

**Fig. 1:**
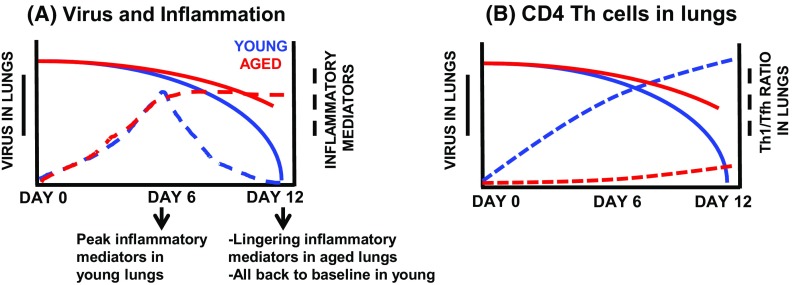
Summary of young and aged responses to influenza infection. Young (2–3 mo) and aged (18–20 mo) male C57BL/6 mice were infected with a sublethal dose of H1N1 influenza. Responses were measured at time points post infection. Results summarized from original published data (Lefebvre et al. 2016b). **a** Virus quantitation and inflammatory mediators (cytokines and chemokines) were assessed in lung tissue and bronchiolar lavage fluid (BAL), respectively. **b** Virus in lungs graphed with the ratios of T_H_1 to Tfh CD4 T cells in the lungs on days 6–12 of infection

Expression of other transcription factors, while in the draining lymph node or later after trafficking to the lung, promote the differentiation into other CD4^+^ T helper (T_H_) subsets. Within the lung, type 1 helper CD4^+^ T cells (T_H_1), distinguished by upregulated transcription factor T-box expressed in T cells (T-bet), secrete interferon-γ (IFN- γ) along with interleukin (IL)-2 at the site of infection. This, along with other chemokines, promotes recruitment of macrophages as well as proliferation of CD4^+^ and CD8^+^ T cells. Additionally, in conjunction with their helper functions, CD4^+^ T cells maintain their own ability to directly lyse virally infected cells as cytotoxic CD4^+^ T cells (T_H_CTL) following up-regulation of the transcription factor eomesodermin (Brown et al. 2006). Further, cytokines secreted by T_H_1 cells enhance T_H_CTL activity. As depicted in Fig. 1a, the peak viral load is approximately 4-6 days post infection in young mice, with a coinciding peak of inflammatory mediators at 6 days post infection. After this peak, both inflammatory mediators and virus in the lungs are reduced until full clearance is reached by approximately day 12 post infection in young mice (Lefebvre et al. 2016b).

We have recently examined the ratio of T_H_1 to T_FH_ in the lungs following flu infection (Lefebvre et al. 2016b). As shown in Fig. 1b, there is about a 2:1, T_H_1:T_FH_ ratio 6 days post infection, increasing to 3:1 by day 12. While the ratio beyond day 12 post infection remains to be seen, it is clear that not only the quality but also quantity of CD4^+^ T_H_ cells is also important during infection. Collectively, in normal young mice, these T_H_ subsets work to promote tightly regulated inflammatory responses at various times throughout infection. This is important to effectively eliminate virally infected cells through cell mediated responses, while controlling inflammation and subsequently promoting a healing and recovery phenotype after virus has been cleared.

Although the majority of CD4^+^ T cell effector functions have been demonstrated using mouse models due to the limited ability to study *in vivo* human CD4^+^ T cell responses, it is important to recognize that many aspects of CD4^+^ and CD8^+^ T cell functions have been corroborated in humans as well (McElhaney et al. 2006). The presence of CD4^+^ and CD8^+^ T cells in the blood following vaccination has been shown as a more accurate readout of vaccination efficacy and the ability to respond to flu virus. A study by McElhaney et al. (2006) demonstrated that the effector function of CD4^+^ and CD8^+^ T cells from flu vaccinated individuals following *ex vivo* stimulation with live flu virus could predict how robust flu responses would be following infection. Indeed, those individuals with lower CD4^+^ and CD8^+^ T cell numbers following *ex vivo* stimulation with live flu virus had a higher rate of laboratory diagnosed flu as opposed to those subjects that had higher CD4^+^ and CD8^+^ T cell numbers (McElhaney et al. 2006). This suggests that the number of CD4^+^ and CD8^+^ T cells could be a better correlate of protection from flu infection following vaccination. While human studies have corroborated the findings in murine studies regarding the importance of CD4^+^ T cells to flu vaccine and memory CD4^+^ T cell responses, the importance of the different subsets has yet to be investigated in elderly humans during flu infection.

### Changes in CD4^+^ T cell subsets with aging and their response to influenza infection

Aging leads to a multitude of changes in the response to flu infection. While the effect of aging on CD8^+^ T cell responses to flu has been extensively studied (Brown et al. 2006; Ely et al. 2007; Hufford et al. 2015), the impact on CD4^+^ T cells is less well understood. Since CD4^+^ T cells are crucial for the plethora of responses to flu as outlined above, age-related changes in CD4^+^ T cells have vast effects on flu immune responses. This is of great clinical importance since flu infection can also increase the risk for other opportunistic and secondary bacterial infections to occur in susceptible aging populations in both mouse models and humans (Lefebvre et al. 2016b; Haynes et al. 2012; Joseph et al. 2013).

Previous in vivo and in vitro studies have shown that age-related changes in murine CD4^+^ T cells influence a variety of functions. The ability of the T cell receptor (TCR) to signal is impaired with aging as demonstrated by reduced immunological synapse formation (Tamir et al. 2000; Garcia and Miller 2001, 2002). In addition, there are defects in activation, differentiation, and proliferation of CD4^+^ T cells from aged mice [reviewed in Haynes and Eaton (2005)]. CD4^+^ T cells also have a reduced capacity to produce IL-2 upon antigenic stimulation with age (Linton et al. 1996) and to provide cognate help to B cells, thus impacting humoral immunity (Eaton et al. 2004). Along with these T cell-intrinsic differences, we have shown that the murine aged microenvironment also negatively impacts CD4^+^ T cell function. Young CD4^+^ T cells when transferred into aged hosts demonstrated a reduction in recruitment, proliferation and differentiation when compared to donor CD4^+^ T cells transferred into young hosts (Lefebvre et al. 2016b). Thus, it is clear decrements in CD4^+^ T cells with aging is multifaceted with both intrinsic and extrinsic factors involved, however mechanisms involved in extrinsic influences remain to be elucidated.

Interestingly, the total number of CD4^+^ T cells in the lungs after flu infection is not different between young and aged mice, however, the ratio of T_H_ subset distribution is impacted by aging (Lefebvre et al. 2016b). Young mice have nearly twice as many T_H_1 cells (indicated by transcription factor T-bet) as T_FH_ (indicated by transcription factor BCL6), which is necessary for creating a robust initial inflammatory response in the lung (Lefebvre et al. 2016b). Aged mice not only have a greater proportion of memory cells (T_mem_), but an approximately 1:1 ratio of T_H_1 to T_FH_ in the lungs during flu infection at day 6 which remains constant through day 12 post infection as shown in Fig. 1b. This not only is represented in the overall CD4^+^ population, but also in the flu nucleoprotein (NP)-specific population as well (Lefebvre et al. 2016b).

Additionally, this age-related difference in T_H_ subset distribution holds true when examining subsets using a scheme developed in the Kaech laboratory (Marshall et al. 2011) where CD4^+^ T cell populations can be broken down phenotypically into subsets based on PSGL1 (CD162) and Ly6C expression to indicate T_H_1 (PSGL1^hi^ Ly6C^hi^), T_mem_ (PSGL1^hi^ Ly6C^lo^) and T_FH_ (PSGL1^lo^ Ly6C^lo^). Similarly, using this scheme, both the total CD4^+^ T cell population as well as the NP-specific CD4^+^ T cell population in the lungs skews towards a T_H_1 phenotype in young mice (Lefebvre et al. 2016b). In contrast, aged mice have fewer total and NP-specific T_H_1 a when compared to young. Thus, regardless of the method of phenotyping CD4^+^ T cells, aged mice do not have the same T cell subset differentiation and distribution as young mice following infection. Furthermore, there is a significant delay in the appearance of flu-specific T_H_1 effector CD4^+^ T cells in the lungs following infection of aged mice, which may contribute to slower viral clearance (Lefebvre et al. 2016b; Lanzer et al. 2014).

With regards to T_FH_, aged mice have increased T_FH_ both by percent and number when compared to young mice at base line as well as on days 7, 10, and 12 post infection (Lefebvre et al. 2016a, b). The finding of more T_FH_ in aged mice may seem counterintuitive, since it is known that aged mice have less robust humoral responses (Eaton et al. 2004; Lefebvre et al. 2016a). It is important to note, however, that despite an increase in T_FH_ cells, the aged T_FH_ do not appear to be as functional and provide reduced levels of help to B cells when compared to young T_FH_. Additionally, within the aged T_FH_ population there is an increase in T follicular regulatory cells (T_FR_) which can function to inhibit T_FH_ helper activity within the germinal center (Lefebvre et al. 2016a). Therefore, the increase in T_FH_ in aged mice does not actually contribute to improving the immune response.

The initial kinetics and clearance of flu virus is indicative of recovery from flu infection (Lefebvre et al. 2016b; Kanegai et al. 2016). Flu infection is a much more severe disease with aging exhibiting slower viral clearance and significantly greater weight loss, a marker of pathogenicity in mouse models (Lefebvre et al. 2016a, b). Inflammatory mediators, including cytokines and chemokines, linger in the bronchiolar lavage fluid (BAL) of aged mice long after they have returned to baseline in young mice (Fig. 1a). By day 12 post-infection, young mice have cleared the virus, resolved inflammation and have returned to their pre-infection weight. In contrast, viral copy number in aged lungs remain elevated at this time point and inflammation has not yet been resolved (Lefebvre et al. 2016b). We hypothesize that the differences in the generation of lung-homing T_H_1 effectors as well as T_FH_ cells and antibody production likely contribute to this dysregulated response to infection. While aging impacts both T_H_ subsets, aged mice exhibit enhanced T_FH_ and reduced T_H_1 generation, as mentioned previously (Lefebvre et al. 2016b). It remains to be investigated whether this preference for T_FH_ differentiation is intrinsic to aged CD4^+^ T cells or is induced by the aged microenvironment. More research is necessary to determine the cause and consequence of this altered differentiation.

Examination of human peripheral blood cells has allowed us to study the humoral responses as well as the circulating cellular responses to flu with age. It has been demonstrated that older adults have a CD4^+^ T cell compartment skewed towards mostly regulatory, memory, and Type 2 like (T_H_2) helper T cells at baseline as well as following flu vaccination (McElhaney et al. 2016; McElhaney et al. 2013). While these subsets may be important in the resolution of flu infection, the reduction in the initial effector response could be a driving force leading to poor viral clearance resulting in much longer recovery time, increased lung damage, and potentially increasing the risk of secondary bacterial infections. While we and others have begun to more deeply examine the mechanisms, both intrinsic and extrinsic, affecting this differentiation, much remains to be investigated. It is essential to determine these mechanisms first in order to begin pre-clinical development of therapeutics aimed at improving the aged response to flu infection.

## Future perspectives

### Potential factors resulting in altered differentiation

It has been shown that TCR repertoire contracts with age and there is a reduced ability to generate new antigen-specific CD4^+^ T cells (Lanzer et al. 2014). Taken together with studies suggesting diminished function, antigen presentation, as well as co-stimulatory molecule and Toll-like Receptor (TLR) expression on APCs (Shaw et al. 2013), it is possible to suggest that this dysregulated differentiation is in part due to defects in CD4^+^ T cell priming. Whether this is a result of TCR or APC functionality and/or signaling, is the question. A more recent study suggests that polarization of CD4^+^ T cells to either T_FH_ or non-T_FH_ is also dependent on TCR-peptide and MHC Class II interactions (Knowlden and Sant 2016). The plasticity of T_FH_ and T_H_1 differentiation has been studied largely in the context of cytokines, antigen presentation by DCs and B cells in the lymph node follicles, as well as co-stimulation [reviewed in (Knowlden and Sant 2016)]. It is possible, however, that in addition to changes in the presence of cytokine and chemokines, which contribute to the overall microenvironment, the limited T cell repertoire and/or the ability for APCs to process and present certain peptides with age influences this shift in CD4^+^ T cell differentiation.

Deficits in APC function have been documented with age (Tamir et al. 2000; Shaw et al. 2010, 2013). As APCs age, they show reduced expression of both co-stimulatory molecules and MHC Class II on their surface (Shaw et al. 2010). Synapses formed between the CD4^+^ T cell receptor and APC MHC molecules are also influenced by aging, showing reduced formation and strength leading to poor CD4^+^ T cell differentiation (Marko et al. 2007; Tamir et al. 2000). It is possible that these alterations in APCs could lead to altered differentiation of CD4^+^ T cells during flu with age.

Another possible factor that might contribute to dysregulated CD4^+^ T cell subset differentiation with age is senescence, which is an irreversible state of cell cycle arrest, in the surrounding tissue cells or immune cells themselves. Senescence has been proven to drive many age-related pathologies (Baker et al. 2011), although it is also crucial to wound healing (Demaria et al. 2014). Senescence can occur in cells for a variety of reasons, including DNA damage, telomere dysfunction, and the expression of oncogenes (Rodier and Campisi 2011). Although senescent cells are in a state of irreversible cell cycle arrest, they do remain metabolically active (Rodier and Campisi 2011) and secrete factors coined the “senescence-associated-secretory-phenotype” (SASP) which includes, but is not limited to, inflammatory interleukins, such as IL-1 and IL-6, many different chemokines, insulin-like growth factors, soluble factors, and other molecules that when chronically secreted can negatively affect the surrounding tissue and microenvironment (Coppe et al. 2010). This in part, is to recruit macrophages and natural killer cells to mediate phagocytosis and self-elimination (Rodier and Campisi 2011). However, with aging, senescent cells are not eliminated efficiently resulting in dramatic accumulation over time (Coppe et al. 2010; Demaria et al. 2014; Baker et al. 2011). The SASP can also cause surrounding and otherwise healthy cells to, in turn, become senescent and is hypothesized to exponentially increase this inflammatory and damaging milieu. It is likely that the accumulation of senescent cells with age and their corresponding inflammatory environment could, even further, play a detrimental role in the function of aged CD4^+^ T cells preventing them from generating a robust anti-viral response. Indeed, the altered cytokine milieu caused by senescent cells may drive the aberrant CD4^+^ T cell differentiation evident during flu infection in aged individuals.

Another potential possibility is that CD4^+^ T cells themselves could become senescent with age, contributing to the intrinsic decrements. It is known that with age, CD4^+^ T cells have reduced production of IL-2, reduced proliferation, and diminished effector functions. Although CD4^+^ T cells have not been examined in the context of cellular senescence specifically, aged CD4^+^ T cells have higher expression of exhaustion markers such as programmed cell death protein 1 (PD-1) (Lefebvre and Haynes 2012). It is important to consider, however, that senescence of CD4^+^ T cells may only be seen in memory CD4^+^ T cells as naïve CD4^+^ T cells and effector CD4^+^ T cells have a rapid turnover, expanding and contracting rapidly following an infection.

Thus, there are several possibilities that can explain how CD4^+^ T cell effector function and differentiation are affected by age. As with many other aspects of aging, there is a very strong possibility that each scenario may simultaneously play a role in age-related CD4^+^ T cell decrements since aging effects many facets of the body and nearly all cellular compartments. Future experiments to address the direct and indirect impacts of senescent cells as well as the SASP on CD4^+^ T cell function and differentiation will be integral in better understanding the mechanism of age-related deficits in CD4^+^ T cell differentiation.

### Manipulating aged CD4^+^ T cell differentiation to improve responses to influenza infection and vaccination

One approach to improve CD4^+^ T cell subset differentiation involves manipulation of the aged microenvironment. It is clear that the aged microenvironment, at least in part, is responsible for diminished CD4^+^ T cell responses (Lefebvre et al. 2016b). It is possible, and likely, that senescent cells and inflammation induced by senescent cells contribute to this disruptive microenvironment that impairs CD4^+^ T cell differentiation, quality, and function. Studies performed by the Kirkland laboratory have shown that elimination of senescent cells has a profound positive impact on age-related diseases (Baker et al. 2011). Thus, it is possible that elimination of senescent cells may also improve CD4^+^ T cell responses to flu infection in older individuals. While the area of senolytics requires much more research prior to translation into humans, it does seem like a promising approach to improve many immune decrements with age. It is possible that any one of these techniques or modifications can elicit a robust CD4^+^ T cell response with a better balance between the effector subsets.

Data suggests that declining humoral and cellular immune responses with age might be contingent upon impaired T helper function. Thus, manipulating CD4^+^ T cell subset differentiation through various approaches may result in more effective viral clearance and faster recovery following infection and/or more robust vaccination responses in the elderly. Pre-clinical research is underway to determine if novel vaccination strategies, such as changing adjuvant composition, including co-stimulatory molecules or cytokines, can boost the CD4^+^ T cell response in aged mice to generate a stronger T_H_1 population and better functional quality of the T_FH_ population after infection (Baldwin et al. 2018). Additional research has focused on employing other target molecules (such as focusing on influenza nucleoprotein instead of hemagglutinin) to enhance aging T cell responses and enhance heterosubtypic influenza immunity (McElhaney et al. 2016).

## Conclusion

Although mechanisms of aberrant CD4^+^ T cell differentiation with aging remains to be determined, it is clear that this plays a strong role in poor flu infection responses in older individuals. While many steps involved in CD4^+^ T cell priming and activation are impaired with aging, it has not been determined yet if one stage is more indicative of poor CD4^+^ T cell function over another, or if, in fact CD4^+^ T cell decrements can be overcome at all to significantly improve the aged response to flu. Research suggests that manipulating vaccine strategies or the extrinsic environment could potentially promote better functioning CD4^+^ T cells with aging, however translation to humans is not clear. It is possible that a combination of preventative and prophylactic strategies will need to be used to effectively protect the elderly population from flu related morbidity and mortality.
